# The Relations Between Recognition Disorders of Familiar People and Other Unique Entities in Patients with Semantic and Behavioral Variants of Right Frontotemporal Degeneration: A Review of Single-Case Studies

**DOI:** 10.3390/brainsci16070705

**Published:** 2026-06-30

**Authors:** Guido Gainotti

**Affiliations:** Institute of Neurology, Università Cattolica del Sacro Cuore, Fondazione Policlinico A. Gemelli, IRCCS, 00168 Rome, Italy; guido.gainotti@unicatt.it; Tel.: +39-06-30156435

**Keywords:** familiar people recognition disorders, unique entities, right frontotemporal degeneration, ‘semantic’ and ‘behavioral’ variants, prosopagnosic and semantic disorders

## Abstract

**Highlights:**

**What are the main findings?**

- Recognition disorders affecting known people and other ‘unique entities’ (UEs; e.g., famous buildings or landmarks) are frequently observed in the ‘semantic’ and ‘behavioral’ variants of right frontotemporal degeneration (rFTD).
- In the first stages of the ‘semantic’ variant, these disorders are often modality-specific, affecting only faces and pictorial forms of ‘UEs’, whereas in the ‘behavioral’ variant, they are often multimodal, affecting ‘faces and names’ and ‘verbal and non-verbal forms’ of UEs as early as the first assessment.

**What are the implications of the main findings?**

- These differences between semantic and behavioral variants can be due to the different evolutional stages at which patients are examined or due to the different loci of lesions in these two variants of rFTD.
- Careful longitudinal studies are needed to clarify this issue.

**Abstract:**

Introduction: An association between recognition disorders of familiar people and other unique entities (UEs), such as famous buildings, is often reported in patients showing a right variant of frontotemporal degeneration (FTD). However, the clinical context and the modality-specific or semantic nature of these disorders have not been clarified by group studies of these patients. Aims of the study: Since a recent consensus statement from the International Working Group on FTD has called for the clarification of these issues, I undertook a review of single-case reports that explored this issue. This review allowed me to identify 11 papers reporting patients affected by a ‘semantic’ or a ‘behavioral’ variant of right FTD. Results: A detailed analysis of these patients suggested the following: (a) the incidence of this association is similar in the ‘semantic’ and ‘behavioral’ variants of right FTD; (b) this association is not systematically observed in patients with a ‘prosopagnosic’ form of the ‘semantic variant, whereas it is more frequent in the ‘properly semantic’ and in the ‘behavioral’ variants; and (c) in these two last groups of patients, the association between poor recognition of familiar people and of other unique entities is usually observed both in the verbal and in the pictorial modalities. Discussion: These results seem to indicate that recognition defects concerning both familiar people and other UEs are due to modality-specific and semantic disorders mainly affecting the right hemisphere’s pictorial knowledge.

## 1. Introduction

Famous people, along with famous buildings, places, and landmarks, are referred to as “unique entities” (UEs) due to the unique nature of the knowledge that we have about them. In their model of brain processes underlying lexical retrieval, Damasio et al. [1,2] suggested that UEs may have a special neural representation in the brain and that the retrieval of corresponding lexical information may be mediated not only by language-related structures, but also by intermediary regions called “convergence zones”. Within this framework, Tranel et al. [3,4] claimed that focal anterior temporal lobe (ATL) damage leads to deficits in the identification and retrieval of information about UEs, mainly concerning known faces and landmarks or buildings. This could result from the fact that both familiar faces and visual representations of UEs are processed through the ‘ventral stream of visual processing’ [5], which connects the primary visual cortex with the ATLs. Within this cortical pathway, face perception is selectively worked out in the fusiform face area [6] on the lateral side of the fusiform gyrus. The resulting structural descriptions of known faces [7] are subsequently connected to the corresponding emotional and memory information within the ATLs [8], and finally, information concerning the face, voice, and name representations of known persons converges in corresponding multimodal semantic representations [7,9].

The modality-specific disruption of face representations is labeled ‘prosopagnosia’, whereas the disruption of multimodal representations of a known person produces a (more or less specific) semantic disorder (see [9] for review). A similar but less specific process is carried out for the visual representations of UEs. In accordance with this model, Damasio et al. [10] suggested that the convergence zones for visual UEs lie within the right temporal pole, whereas the left temporal pole contains convergence zones regarding conceptual knowledge about verbal UEs. This model was supported by Tsukiura et al. [11] in a training study in which participants learned the names and occupations of people identified through their photographs, because the authors showed that the left ATL links information about persons to their proper names, while the right ATL links information about persons to their corresponding faces. The major role of the left ATL in the processing of verbal forms of UEs was confirmed by neuroimaging studies (e.g., [12,13,14]), which reported that the left ATL is sensitive to the naming or retrieval of verbal information about famous people and landmarks. Thus, Grabowski et al. [13] found that the left temporal pole was significantly activated when subjects named known people and landmarks, and Tranel [14] showed that patients with left temporal polar lesions performed worse than patients with right temporal polar damage on a ‘person and landmark naming task’. On the other hand, the major role of the right ATLs in the processing of non-verbal forms of UEs has been documented by Seidenberg et al. [15] and by Benke et al. [16], who studied the different stages of proper name retrieval in patients with unilateral forms of temporal lobe epilepsy (TLE). Seidenberg et al. [15] showed that patients with left temporal lobe epilepsy (TLE) presented a selective impairment in naming famous faces but performed similar to the controls in face recognition and semantic identification, whereas right TLE patients were consistently impaired in tasks of face familiarity identification and naming. These data were clarified by Benke et al. [16], who showed that the side of seizure onset differentially influenced the stages of name retrieval, because left TLE mainly impaired the lexical–phonological processing of names, whereas right TLE preferentially impaired the perceptual–semantic stage of face recognition.

In the period of time in which many influential investigations (e.g., [17,18,19,20,21,22,23,24]) of recognition disorders for faces and for other kinds of UEs had been conducted in patients with lesions involving the right ATL, these studies had been motivated by the wish to analyze the ‘functional architecture’ of person recognition disorders. However, in more recent times, interest in these studies has greatly increased due to the acknowledgement that face recognition disorders are critically involved in patients affected by a right variant of frontotemporal degeneration (RvFTD), a form of degenerative brain disease that has been intensively investigated in recent years. Different forms of familiar people recognition disorders are indeed systematically observed in the right and left variants of this degenerative disease, which selectively involves the ATLs. In particular, patients with right ATL damage usually show an impaired recognition of familiar faces (associative prosopagnosia) linked sometimes to other cognitive defects and very often to emotional and behavioral disorders that can include disinhibition, apathy, and loss of empathy [25,26]. The other less frequent cognitive defects observed in these patients can include the defective recognition of other UEs [18,19] and (less frequently) category-specific disorders for living beings [21,27] and the loss of one’s ability to perceive flavors and odors [28]. For this reason, Josephs et al. [29] have systematically investigated recognition disorders both of known people (prosopagnosia) and of UEs (landmarks) in a group of patients affected by RvFTD. On the basis of clinical, neuropsychological, genetic, and neuropathologic features, these authors identified 20 subjects with right temporal FTD, and within them, distinguished 12 patients diagnosed as having ‘right behavioral’ variants (R.bvFTD) from the other eight diagnosed as having ‘right semantic’ variants (R.semvFTD) of FTD. In these patients, Josephs et al. [29] investigated both ‘prosopagnosia’ and a category of UEs (‘topographagnosia’), defined as a ‘landmark agnosia’, in which patients are unable to perceive and identify familiar buildings and landscapes [30,31]. They showed that, during the first 2 years after onset, recognition disorders of known people (prosopagnosia) and of unique entities (landmark agnosia) are significantly more frequent in the ‘semantic’ FTD group, whereas personality changes and inappropriate behaviors are significantly more common in the ‘behavioral’ FTD group. The voxel-based morphometry (VBM) detected more frontal lobe atrophy in the ‘behavioral’ than in the ‘semantic’ group, which conversely showed greater loss in the right fusiform and lateral temporal lobes. Another imaging characteristic that differentiated the R.semvFTD subjects from the R.bvFTD subjects was that the temporal lobe asymmetry was greater in patients with the ‘semantic’ variant than in those with the ‘behavioral’ variant of FTD. These findings suggested to Josephs et al. [29] that the fusiform gyrus might be the region whose atrophy accounts for both prosopagnosia and landmark agnosia in patients with a right ‘semantic’ variant of FTD. These data are interesting because they suggest that both ‘prosopagnosia’ and ‘topographagnosia’ may be more frequent in the semantic than in the behavioral variant of right FTD, but it is not possible to evaluate if these associated disorders are due to the disruption of a multimodal semantic or an agnosic modality-specific visual mechanism. To evaluate the nature of the underlying mechanism, associations and dissociations between recognition disorders of known people and of other unique entities should be assessed in individual patients, but an assessment was not conducted in Josephs et al.’s [29] group study. Two other reasons could explain why it is very difficult to clarify the mechanism underlying the association between recognition disorders of familiar people and of other UEs in patients with a right variant of FTD. The first reason is the low number of case reports that have investigated recognition disorders of both these categories of UEs in patients with a right variant of FTD. The second reason is the high number of patients for whom person recognition disorders were assessed only through face (prosopagnosia) but not through other (voice and name) recognition modalities. Furthermore, when these multimodal assessments were conducted for the same individuals, inconsistent results were sometimes obtained in patients with right semvFTD. Some authors (e.g., [9,32,33]) have, indeed, shown that the distinction between modality-specific (prosopagnosic) and multimodal (semantic) face recognition disorders can be more complex than this simple dichotomy suggests. The usefulness of clarifying the nature of the association between known people and other UE recognition disorders has been stressed in recent times by Ugulut et al. [34], who have stated that, due to the lack of a full clinical characterization and precise terminology, an accurate diagnosis of FTD remains challenging in patients with right ATL damage. According to these authors, one of the issues that requires clarification concerns the relation between the impaired recognition of familiar people and other kinds of UEs shown by these patients. I therefore thought that a careful review of single-case reports that have selectively investigated this issue could be useful to identify the factors underlying recognition disorders of famous persons and other UEs in patients with a right variant of FTD. More specifically, I intended to clarify the following points:

1. Are these recognition disorders much more frequent in patients diagnosed as R semvFTD, as suggested by Josephs et al. [29], or are they equally present in patients with R. bvFTD?
2. Are recognition disorders for known people and other UEs equally frequent in persons with multimodal and in those with modality-specific (prosopagnosic) person recognition disorders?
3. Are the neuro-anatomical correlates of recognition disorders for famous persons and other UEs different in patients with ‘semantic’ and ‘behavioral’ variants of R. FTD?

## 2. Methods

To clarify the above-mentioned points, I took into account all publications reporting single-case studies of patients diagnosed with right variants of FTD that had investigated, from a clinical or neuropsychological point of view, recognition disorders of famous (or familiar) people and of unique entities (famous landmarks, places, and monuments) that could be identified on the basis of their visual features. With this aim in mind, I used PubMed and Web of Science to search for studies that included case reports relevant to this issue. The search keywords included terms related to person recognition disorders (‘face recognition disorders’ OR ‘prosopagnosia’ OR ‘person recognition disorders’) AND keywords related to unique entities (unique entities OR famous landmarks OR famous monuments).

The flow diagram of the review process was as follows.

In the identification stage, 23 records were identified from PubMed and/or Web of Science, and 10 additional records were identified from the references of the obtained articles. In the screening stage, papers in which unique entities could not be recognized through the visual modality (e.g., ‘famous songs’) were excluded because visual (‘face’) recognition is the recognition modality systematically studied in patients with a right variant of FTD and we intended to include only studies in which UEs are recognized through the same modality in this review. Furthermore, two papers in which disorders of famous people and landmarks recognition had been repeatedly evaluated with similar neuropsychological tasks were included as independent studies, because they allowed us to specifically evaluate the influence that disease progression could have on the association between famous people and famous UEs. On the basis of these criteria, 22 of the previously identified papers were removed because they concerned patients who had only been evaluated for face recognition disorders or UEs (e.g., famous songs) that could be recognized only through auditory modality or neuroimaging data and did not allow for evaluations of the extension and lateralization of brain atrophy. The remaining 11 publications reported data concerning 9 patients who satisfied our inclusion criteria. Even if the number of papers included in the review was rather small, the quality of these papers and their informative content were very high, as documented by the number of their citations. This number ranged, in fact, from 19 to 560, with a mean value of 143 (the number of citations obtained by each paper, according to Scopus, is reported in Table 1). For this reason, it was decided that the informative content of the review could be relevant despite the small number of papers satisfying the inclusion criteria.

### Data Taken into Account for Each Patient Included in the Present Review

The following criteria were used for the tabulation of patients included in this review:

(a). *Distinction between patients diagnosed as ‘behavioral’ vs ‘semantic’ variants of right FTD*.

Patients were diagnosed as R.bvFTD if behavioral disorders were the first symptom or the main clinical disorder shown by the patient. They were, in contrast, diagnosed as R. semvFTD if face recognition disorders were the first symptom and behavioral disorders were not at the forefront of the symptomatology shown by the patient. The presence or absence of the neuroradiological involvement of the right frontal lobe was also taken into account, because, according to many authors (e.g., [29,35,36,37]), atrophy of the right frontal lobe could be a marker of R.bv FTD.

Furthermore, in patients diagnosed as R. semvFTD patients, two different subgroups of subjects showing, respectively, a modality-specific (‘prosopagnosic’) and a multimodal (‘properly semantic’) form of person recognition disorders were distinguished. This distinction between associative prosopagnosia and multimodal semantic person recognition disorder is based on the fact that, in associative prosopagnosia, the recognition of familiar people is impaired only through the face modality, whereas in multimodal semantic person recognition disorder, it is impaired through all the recognition modalities (face, voice, and name).

This further fragmentation of the small number of patients included in the present review into two different subgroups could have both negative and positive effects. On one hand, it is impossible to evaluate the significance of our results with statistical methods, but on the other hand, the small groups of patients for whom results were obtained were homogeneous. It was decided that this second aspect could be at least as relevant as the first one in clarifying the aims of the review.

For each case included in the review, the following data were taken into account:

(b). *Main clinical data*: Age at onset, initial clinical symptoms, and clinical course of the disease were taken into account;
(c). *General neuropsychological investigations*: General cognitive, language, attentional, and memory disorders that could explain part of the symptomatology were considered. Particular attention was paid to general visual recognition disorders that could explain modality-specific recognition disorders for faces and pictorial representations of other UEs and to language disorders that could point to the functional involvement of the left temporal lobe;
(d). *Person recognition* was investigated, taking into account both the main channels (face, voice, and name) and the different stages (familiarity, identification, and naming) of the familiar people recognition process and making a distinction between modality-specific (‘associative prosopagnosia’) and multimodal (verbal and non-verbal) person recognition disorders;
(e). *Recognition of unique entities*: In the evaluation of UE recognition disorders, we tried to use (when possible) criteria similar to those followed for famous people recognition disorders, namely the distinction between modality-specific and multimodal recognition disorders and the stages of the UE recognition process (familiarity, identification, and naming);
(f). *Anatomical lesions*: The localization of atrophy within the right temporal lobe, the presence of frontal lobe involvement, and the degree of asymmetry of ATL atrophy were taken into account.

## 3. Results

All data that were taken into account to clarify the relations between recognition disorders of familiar people and of other unique entities in patients with right variants of frontotemporal dementia are reported in Table 1.

**Table 1:** Data taken into account for every patient included in the review.

**A. Patients diagnosed as ‘semantic’ (‘prosopagnosic-semantic’ or ‘properly semantic’) variants of right FTD.**	
**1. VH**	[22] (Evans et al., 1995): 560 citations.
Diagnosis	Prosopagnosic–semantic right-sided variant of FTD.
Main clinical data	VH, a 68-year-old right-handed woman, showed a progressive difficulty recognizing people by their faces. Her knowledge of people was initially much better from their name than from their face, but this difference progressively disappeared. Time from onset: 24 months; disease severity: mild.
General neuropsychological investigations	No disorders of intelligence, language, memory, or attention. General visual recognition: some problems were found on a flower recognition task. Language: within normal limits.
Person recognition	Face discrimination was intact, but VH was moderately impaired on familiarity judgement and severely impaired on face identification. Voice recognition was not formally tested. Knowledge of people from names was originally much better than from faces. The person recognition defect was considered as a modality-specific form of associative prosopagnosia that progressed to a multimodal person recognition disorder more severe for faces than for names.
Recognition of unique entities	VH showed a striking dissociation between her difficulty in recognizing faces and her spared capacity to identify pictures of flowers and of other unique entities, such as buildings. The dissociation between faces and other unique visual entities was underlined by the authors.
Anatomical lesions	SPECT scanning indicated marked hypoperfusion in the anterior-lateral right temporal lobe, and MR imaging revealed atrophic changes involving the right temporal lobe with relative sparing of the superior temporal gyrus and hippocampal region.
**2. VH**	[38] (Kitchener and Hodges, 1999): 39 citations.
Main clinical data	At the time of this second examination, VH had shown a progressive worsening of his person recognition disorders, but continued to live alone with minimal support, though exhibiting problems with anterograde memory and a slight spatial disorientation. Time from onset: 60 months; disease severity: mild.
General neuropsychological investigations	No significant decline was found in her general intellectual abilities or in her working memory. General visual recognition: some problems were found on a visual recognition task. Language: within normal limits.
Person recognition	At the time of this assessment, VH performed at floor level on knowledge of famous people, whether tested using faces or names, familiarity feelings, identification, or naming. This disorder was considered as a severe multimodal defect of known person recognition.
Recognition of unique entities	VS was at or near floor level on tests of ‘Famous events’ identification, irrespectively of the visual (photographs) or verbal modality of access and of the recall, familiarity, or recognition modality required. This disorder was considered as part of a multimodal semantic defect.
Anatomical lesions	At the time of this assessment, an SPECT scan revealed marked hypo-perfusion of the right temporal lobe, with minor extension into the left temporal lobe and mildly reduced frontal uptake bilaterally. ATL atrophy was strongly prevalent on the right side.
**3. Emma**	[39] Gentileschi et al. (2001): 152 citations.
Main clinical data	Emma, a 60-year-old, right-handed woman, showed slowly progressive difficulties in the recognition of familiar people. This defect was not overcome by extra-facial visual information such as listening to the voice of or hearing the correct name of an unrecognized familiar person. Emma also presented slight changes in her behavior. Her person recognition disorder worsened very slowly. Time from onset: 48 months; disease severity: mild.
General neuropsychological investigations	There were no signs of general cognitive deterioration or memory and attentional problems. General visual recognition: there was no evidence of visuo-perceptual or visuospatial deficits. Language: Her language was normal, except for rare instances of anomia.
Person recognition	On tasks of face recognition, she obtained good results on apperceptive tests but was severely impaired on face familiarity and identification tasks. She was also unable to identify the voice of highly familiar speakers and moderately impaired in recognizing familiar persons from their names. These recognition disorders were considered a multimodal disorder of person recognition.
Recognition of Unique Entities	Emma was given famous Italian pop songs with which she had been familiar and asked to state whether those pieces of music were familiar or not, and, if they were familiar, the title of the song or the name of the singer. She never managed to tell either the title of the song or the name of the singer and reported a feeling of familiarity for less than half of these pieces of music. Some degree of familiarity could therefore be achieved, but a semantic identification was impossible.
Anatomical lesions	An MRI showed atrophy that encroached almost only upon the anterior and inferior aspects of the right temporal lobe and was marginal on the left side. These data were confirmed by an SPECT scan which showed a hypoperfusion mainly affecting the right temporal region.
**4. FG**	[40] (Joubert et al., 2003) 155 citations.
Diagnosis	Prosopagnosic–semantic right-sided variant of FTD.
Main clinical data	FG, a 71-year-old, right-handed man, presented with a slowly progressive deterioration in his ability to recognize faces of familiar and famous persons, contrasting with the relative preservation of other cognitive domains. He could compensate for his person recognition defects by hearing their voices or their names. Along with his face recognition deficit, FG also exhibited mild visual agnosia. Time from onset: 60 months; disease severity: mild.
General neuropsychological investigations	No disorders of intelligence, language, memory, or attention. General visual recognition: there some problems on post-perceptual visual recognition tasks. Language was normal, except for rare instances of anomia.
Person recognition	On tasks exploring face recognition, a mild impairment was found in face configurational processing. Face familiarity was moderately impaired, and face identification and naming were severely impaired. Name recognition was normal, and voice recognition was clinically intact. The person recognition defect was considered to be due to impaired configurational processing of visually complex entities in the absence of semantic deficits.
Recognition of unique entities	FG could name and recognize only the photos of a few (about 30%) famous monuments that he could correctly identify upon the presentation of their names, but these defects seemed to be due to deficits in their perceptual identification. Similar asymmetries were found when investigating their recognition of famous events.
Anatomical lesions	MRI imaging revealed a marked grey matter volume reduction in the fusiform gyrus, the para-hippocampal gyrus in its posterior part, and the hippocampus bilaterally, although the atrophy was far more extensive in the right hemisphere than in the left.
**5. FG**	[41] (Joubert et al., 2004): 55 citations.
Diagnosis	Properly semantic right-sided variant of FTD.
Main clinical data	At the time of this second examination, VH showed a progressive worsening of his person recognition disorders, but they continued to live alone with minimal support, though they were exhibiting problems with their anterograde memory and slight spatial disorientation. Time from onset: 84 months; disease severity: moderate.
General neuropsychological investigations	When compared with the initial evaluation, the patient had lost considerable knowledge about familiar persons and famous celebrities. There were no behavioral changes, but the patient’s general intellectual, memory, and cognitive abilities had clearly worsened. General visual recognition: there were some problems on post-perceptual visual recognition tasks. Language was normal, except for rare instances of anomia.
Person recognition	The patient’s apperceptive prosopagnosia had evolved to a cross-modal person recognition deficit. He not only failed to recognize familiar faces but did not feel these faces were familiar and could not identify or name them. He also failed to name or identify 40 celebrities from their photographs and from their names, showing a global (semantic) person recognition defect.
Recognition of Unique Entities	The same categories of unique entities initially affected by his visuo-perceptual disturbances (i.e., famous public events and famous monuments) were affected two years later by his semantic deficit, to a greater extent than other categories.
Anatomical lesions	At the time of this assessment, an SPECT scan revealed marked hypo-perfusion of the right temporal lobe, with minor extension into the left temporal lobe and mildly reduced frontal uptake bilaterally. ATL atrophy was strongly prevalent in the right side.
**6. MT**	[42] (Nakachi, et al., 2007): 19 citations.
Diagnosis	Prosopagnosic–semantic right-sided variant of FTD.
Main clinical data	MT, a 68-year-old, right-handed, highly educated woman, was referred to the hospital because of subtle involuntary movements of the trunk, but her son reported that she could not learn the faces of recent acquaintances. She had an MRI that revealed the atrophy of the right ATL. Time from onset: 36 months; disease severity: mild.
General neuropsychological investigations	No general cognitive deterioration or disorders of memory and executive functions. General visual recognition: some problems on visual recognition tasks within and across categories. Language: within normal limits.
Person recognition	MT presented deficits in tests assessing their familiarity and recognition of faces of famous people and family members, but was able to recognize familiar people by their voice and could access information about famous people when hearing the person’s name. The person recognition defect was considered as a modality-specific form of associative prosopagnosia.
Recognition of unique entities	She was given the Visual Remote Memory Test, which consists of photographs of famous people’s faces and of famous scenes. Her errors were restricted to photographs of faces, whereas all her responses to the photographs of famous scenes were correct.
Anatomical lesions	Bilateral atrophy of the anterior temporal lobes was greater on the right side, where the atrophy extended to the fusiform gyrus, though the left gyrus was well preserved.
**7. MD**	[43] (Busigny et al., 2009). 67 citations.
Diagnosis	Properly semantic right-sided variant of FTD.
Main clinical data	MD is a 71-year-old right-handed woman who presented a progressive defect in the recognition of familiar faces (prosopagnosia), associated with difficulties in retrieving words and progressive behavioral disorders. The patient did not have defects in her general memory or spatial-temporal orientation and had good awareness of her deficits. Time from onset: 24 months; disease severity: moderate.
General neuropsychological investigations	No general cognitive impairment; no impairments in episodic memory, executive functions, or visual spatial abilities. General visual recognition: some problems on visual recognition tasks within and across categories. Language: severely impaired on oral production and comprehension and in written comprehension.
Person recognition	Normal results on tasks testing her discrimination of faces and objects, but she had a massive impairment in tasks of face familiarity, recognition, and naming. She performed better but still pathologically on similar tasks when the people’s names were provided. These person recognition defects were considered to be a multimodal semantic disorder that was more severe for faces than names.
Recognition of unique entities	MD showed an extremely low accuracy in recognition (from photos and from names) of famous places previously well known to the patient. These disorders were, however, less severe than those concerning people recognition.
Anatomical lesions	A structural MRI showed clear atrophy of the anterior parts of the temporal lobes with a right lateral dominance. A positron emission tomography (PET) examination showed hypoperfusion of the anterior parts of the right temporal lobe and, to a lesser extent, the right frontal lobe.
**8. Co**	[44] (Gainotti et al., 2003). 261 citations.
Diagnosis	Properly semantic right-sided variant of FTD.
Main clinical data	CO, a 49-year-old right-handed man working in a bank, showed a selective defect in the recognition of familiar people, followed by a mild deterioration in his social activities, with an increased reduction in the number and quality of social contacts. The clinical course was characterized by mild disease progression. Time from onset: 12 months; disease severity: moderate.
General neuropsychological investigations	He was repeatedly tested from June 199 to February 2022, showing very mild changes on general neuropsychological tests, with no general cognitive impairment or disorders of episodic memory, attention, and executive functions. General visual recognition: no problems in visual perception or visual-spatial abilities. Language: semantic–lexical disorders on semantic word fluency and object-naming tasks.
Person recognition	CO showed no defects in face discrimination but a moderate impairment in face familiarity and a severe impairment in face identification and naming. Voice recognition was as impaired as face recognition, whereas name recognition was normal. These recognition disorders increased with time and were considered as a multimodal disorder of person recognition but restricted to the non-verbal (face and voice) modalities.
Recognition of unique entities	CD identified items belonging to semantic categories much better than those considered as ‘unique entities’. Within the latter, he was more impaired in recognizing famous people than famous towns or monuments. He also obtained very good scores in naming famous towns from definitions, suggesting a greater non-verbal disorder concerning faces more than pictures of famous UEs.
Anatomical lesions	An MRI showed a clear atrophy of the antero-inferior parts of the temporal lobes, greater on the right side, and questionable atrophy of the frontal lobes. An SPECT scan showed a severe hypo-perfusion restricted to the anterior parts of the right temporal lobe.
**B. Patients diagnosed as ‘behavioral’ variants of right FTD.**	
**9. MF**	[21] (Barbarotto et al., 1995): 180 citations.
Diagnosis	Behavioral Right-sided variant of FTD
Main clinical data	At the year of 54 MF (a successful architect) began to show word- finding problems followed by person recognition difficulties and behavioral disorders (very obsessive and concerned with religious themes). The clinical course of the disease was characterized by a slow development of behavioral (obsessional rituals, disinhibition) and eating disorders. Time from onset: 120 months; disease severity: severe
General neuropsychological investigations	No disorders of intelligence, memory, and attention, General visual recognition: some problems on post-perceptual visual recognition tasks. Language: severely impaired semantic memory of living beings.
Person recognition	Face discrimination was intact, but she was moderately impaired on familiarity and judgement and severely impaired on face identification and face naming. Similar but less severe defects were observed giving the names corresponding to the face stimuli. The person recognition disorder was considered as multimodal but more severe for face than for name
Recognition of unique entities	On tasks of visual items recognition MF showed familiarity and recognition disorders for living items. Similar defects were noted in response to verbal questions concerning non-perceptual properties of living entities. Severe disorders were observed asking the patient to identify, naming, and giving relevant architectural information about pictures of famous monuments. Similar but less severe difficulties were detected using verbal material. These UEs recognition disorder were, therefore, multimodal but more severe in the visual than in the verbal modality.
Anatomical lesions	On the MRI the patient presented a severe atrophy of the anterior temporal lobes, more important on the right side, that also in- volved basal neocortex, hippocampus, and para-hippocampal gyri. The cella media of the frontal horn was enlarged on the left side, whereas the ATL atrophy was strongly prevalent of the right side.
**10. JT**	[45] (Gorno-Tempini et al., 2004): 218 citations.
Diagnosis	Behavioral right-sided variant of FTD.
Main clinical data	JT, a right-handed 67-year-old woman, showed behavioral disorders, with loss of empathy and unconcern about others, followed, about one year later, by difficulty in recognizing familiar people through all modalities and by a severe impairment in recognizing foods by their look, flavor, or name. Deterioration of semantic memory extended to the recognition of words and common objects. Time from onset: 36 months; disease severity: severe.
General neuropsychological investigations	JTs showed low average or impaired performance in both verbal and non-verbal memory tests and on speech and language assessment severe naming and comprehension disorders (mainly concerning living beings). Her comprehension of syntactic structures and her reading abilities were preserved.
Person recognition	No difficulty of face discrimination, but moderate impairment on face familiarity and severe impaired on face identification and naming. Voice recognition was also severely impaired (face recognition was not improved by hearing the person’s voice) Name identification was severely impaired. Her person recognition disorder was considered as a semantic multi-modal recognition disorder.
Recognition of unique entities	JT was severely impaired on a category fluency test with animals and complained particularly of not remembering the names of countries and cities that she had visited multiple times.
Anatomical lesions	Voxel-based morphometry (VBM) showed most significant atrophy in the right amygdala/anterior hippocampal complex and col- lateral sulcus, extending to the right insula.
**11. CD**	[46] (Gainotti et al., 2008): 67 citations.
Diagnosis	Behavioral Right-sided variant of FTD
Main clinical data	CD, a 53-year-old right-handed woman, with 8 years of formal education, began to show behavioral disorders and difficulty recognizing familiar people. No difference was found between the different modalities of person recognition., Behavioral disorders and recognition defects rapidly worsened and were followed by stereotypic behaviors, with echolalia and palilalia and by the development of a motor neuron disease time from onset: 18 months’ ‘disease severity’ severe.
General neuropsychological investigations	No cognitive impairment or disorders of episodic memory, attention, and executive functions. General visual recognition: no defects on tasks of visual perception or visual-spatial abilities. Language: abnormal scores on tests of word production (phono logical and semantic word fluency, object, and action naming task).
Person recognition	Not impaired on face discrimination, but impaired on face familiarity and identification. Severely impaired on voice familiarity and identification and on name recognition These recognition defects were considered as a multimodal semantic disorder of person recognition.
Recognition of unique entities	CD identified items belonging to general semantic categories much better than those considered as ‘unique entities’ and, within the latter, was more impaired recognizing famous people than famous towns or monuments.
Anatomical lesions	An MRI showed a clear atrophy of the antero-inferior parts of the temporal lobes, greater on the right side, and of both frontal lobes. Single photon emission computer tomography (SPECT), showed an important hypoperfusion of the anterior and inferior parts of the temporal lobes (a), more severe on the right and extending to the dorsal parts of the right frontal and temporal lobes.

### Analysis of Data Reported in Table 1 for Each Variable Considered in the Present Review

Since one of the main purposes of this review consisted in assessing if recognition disorders for known people and UEs are mainly found in patients diagnosed with ‘semantic’ rather than ‘behavioral’ variants of the right FTD, we have separately reported in the upper part of Table 1 eight papers concerning patients diagnosed as having ‘semantic’ (three ‘prosopagnosic’ and five ‘properly semantic’) variants and, in its lower part data, three patients diagnosed as having ‘behavioral’ variants of the right FTD. Two pairs of papers, namely those regarding patient VH (papers 1 and 2) and patient FG (papers 4 and 5), concerned two patients diagnosed as having ‘semantic’ variants of right FTD, both in the first and in more advanced stages of their disease. In the first examination, these patients (1 and 4) had been considered as ‘prosopagnosic’, whereas in the second examination, they (3 and 5) were judged as affected by a ‘properly semantic’ form of a person recognition disorder. A comparison between the results obtained by the same patients in these two different stages of the person recognition disorder could therefore clarify the role played by intervening factors during the disease’s evolution in the relations between recognition disorders for known people and for other kinds of UEs.

For each subgroup of patients taken into account, the following results were obtained:

*Incidence of recognition disorders for UEs other than known people in different subgroups of patients*: Almost all patients, irrespective of their diagnostic group, showed an association between recognition disorders for known people and for other UEs. The only exceptions were two patients affected by a prosopagnosic–semantic variant of right FTD, namely VH in the first stages of the disease (1), and patient MT (6), who had also shown an impaired recognition of faces but not of other unique visual entities.

*Relations between the modality-specific or semantic nature of the recognition disorders for known people and for other UEs in different subgroups of right FTD patients:* These relations were taken separately into account: (a) in three patients (1-VH, 4-FG, and 6-MT) with a prosopagnosic–semantic variant of right FTD, (b) in five patients (2-VH, 3-Emma, 5-FG, 7-M, and 8-Co) with a properly semantic variant of right FTD, and (c) in three patients (9-MF, 10-JT, and 11-CD) with a behavioral variant of right FTD.

(a) Patients with a prosopagnosic–semantic variant of right FTD: In two of these patients (1-VH and 6-MT), their visual identification of UEs other than known people was intact, whereas in patient 4-FG, both their person recognition defect and their faulty visual identification of famous monuments seemed to be due to modality-specific visual deficits;
(b) Patients with a properly semantic variant of right FTD: In all patients affected by a properly semantic variant of the disease, multimodal recognition disorders were observed both on tasks of familiar person recognition and in the identification of other UEs, even if in some patients (e.g., 7-MD and 8-Co) the visual modality was more affected than the verbal modality;
(c) Patients with a behavioral variant of right FTD: In all patients affected by a behavioral variant of right FTD, both the person recognition defect and the wrong identification of other UEs were multimodal, affecting their recognition of known people and of other UEs across different presentation modalities. However, in patient 9-MF, both recognition disorders were more affected in the visual than in the verbal modality, and in patient 11-CD, their person recognition defect was more severe than their recognition of famous towns and monuments.

*Relations between recognition disorders for known people and other UEs and general disturbances of visual perception or of language disorders*. Some relations between modality-specific visual recognition disorders for known people and other UEs were found in patient 1-VH, 4-FG) and 6-MT, diagnosed as having prosopagnosic–semantic variants of FTD, and in patients 2-VH, 5-FG, 7-MD, and 8-Co, considered to have the properly semantic variant of FTD. A similar prevalence of modality-specific visual over general semantic disorders was observed only in one patient (9-MF) diagnosed as having the ‘behavioral’ variant of right FTD. On the other hand, no semantic–lexical language disorders were observed in the patient diagnosed with prosopagnosic–semantic variants of FTD, whereas language disorders were observed in two patients (7-MD and 8-CO) considered to have properly semantic variants of FTD and in all three patients (9-MF, 10-JT, and 11-CD) diagnosed with ‘behavioral’ variants of right FTD.

*Relations between the anatomical distribution of atrophy and the clinical form of RvFTD or the frequency of recognition disorders for famous persons and other UEs.* No clear relation was found between neuroanatomical and cognitive data. No difference was indeed observed between the degree of asymmetry of the ATL atrophy and the clinical form of RvFTD or the frequency of recognition disorders for famous persons and other unique entities. In fact, only two patients showed atrophy restricted to the right ATL, but they belonged to very different diagnostic categories. One of them was patient 1-VH, diagnosed with a prosopagnosic–semantic variant of right FTD, who showed a selective form of associative prosopagnosia, with intact recognition of other UEs, whereas the other patient was 10-JT, diagnosed with a behavioral variant of right FTD, who showed a multimodal recognition disorder affecting both known people and other kinds of unique entities.

All the other patients showed bilateral atrophy of the ATLs that was more severe on the right side. Two of these patients, namely, 4-FG and 6-MT, had been diagnosed with prosopagnosic–semantic variants of right FTD, and one of them (patient 6-MT) showed a selective form of associative prosopagnosia, without impaired recognition of other UEs. Four other patients (i.e., 2-VH, 3-Emma, 5-FG,7-MD, and 8-Co) were considered to have properly semantic variants of FTD, and the last two patients (9-MF and 11-CD) were diagnosed as having behavioral variants of right FTD.

Analogously, no clear difference was observed between the presence of frontal lobe atrophy and the clinical form of RvFTD or the frequency of recognition disorders for famous persons and other unique entities. A mild extension of atrophy to the frontal lobes was indeed found in two patients (7-MD and 8-Co) with a properly semantic variant and in two (9-MF and 11-CD) with a behavioral variant of right FTD. All these patients showed multimodal recognition disorders of known persons and of UEs.

## 4. Discussion

The results of the present review seem to confirm that recognition disorders affecting famous buildings, places, and landmarks, in addition to familiar people, are frequently observed in patients with RvFTD. Person recognition disorders have been reported more frequently than those affecting other kinds of UEs, but this discrepancy is probably due to the much greater attention paid to disorders affecting persons than to those concerning other UEs. The data gathered in this review seem, however, to show that the relation between these two disorders differs according to the clinical form and to the stage of evolution of the disease. Josephs et al. [29] suggested that this association could be more frequent in patients with a ‘semantic’ than in those with a ‘behavioral’ variant of right FTD, but the data of the present review do not support this statement.

These data seem, in fact, to indicate that the association between these recognition disorders concerns a more advanced stage of the disease’s evolution and the properly semantic nature of the recognition defect than the difference between the semantic and behavioral forms of RvFTD. Disorders of persons and UE recognition were indeed present in most patients reported in the present review, irrespective of their diagnosis with the ‘semantic’ or ‘behavioral’ variant of right FTD. Some differences in frequency and nature of the associated recognition disorders were, on the contrary, found between patients with a ‘prosopagnosic—semantic’ and with a ‘properly semantic’ right variant of FTD. In the first group of patients, person recognition disorders were, in fact, modality-specific and were more frequent and severe than recognition disorders affecting landmarks of monuments. It was only in more advanced stages of the disease, namely, in patients with a ‘properly semantic’ right variant of FTD, that the recognition of other kinds of UEs became more frequent and extended from the pictorial to the verbal modality. This shift in the pattern of impairment was particularly clear when examining the data obtained in the initial and in more advanced stages of the disease in patient VH (1 and 2). This patient showed in the first stages of her disease an associative prosopagnosia that subsequently progressed to a multimodal person recognition disorder, which was more severe for faces than for names. In the first stage of her disease, VH showed a striking dissociation between her difficulties in recognizing faces and her intact capacity to recognize pictures of unique entities, such as buildings. In more advanced stages of the disease, however, the person recognition defect evolved to a severe multimodal deficit affecting both visual (face) and verbal (name) channels of person recognition, and a very similar pattern of impairment was documented for tasks of recognition of other unique entities (‘Famous events’). Very similar dissociations between modality-specific recognition disorders for persons but not for other unique entities (“famous scenes”) were observed in patient MT (6), who was also affected by a prosopagnosic–semantic right-sided variant of FTD, because this patient made recognition errors when looking at photographs of famous faces but not photographs of famous scenes. Finally, a shift in the pattern of impairment similar to that shown by patient VH was also observed in patient FG (4 and 5), who had shown in the early stage of his disease modality-specific recognition disorders affecting both people and other kinds of UEs (famous monuments and events). In more advanced stages of the disease, in fact, both these kinds of recognition disorders evolved from the modality-specific to the semantic level, affecting both the visual and the verbal modalities. Patterns of recognition impairment similar to those observed in patients with a ‘properly semantic’ variant of right FTD were also observed in patients with a ‘behavioral’ variant of the same disease, because in these individuals, recognition disorders were often multimodal and affected the recognition of familiar people and of other UEs. So, in our review, the association between recognition disorders for people and other UEs was not usually present in the earliest (prosopagnosic) stages of the R svFTD but was systematically observed in the more advanced ‘properly semantic’ stages and in the behavioral forms of right FTD. This could be due to several reasons. The first reason could be selection bias: the patients considered in this review may have been studied earlier and in more detail when their semantic disorders were more clearcut than behavioral disorders. This interpretation is consistent with Youles et al. [47]’s observation that patients with predominant behavioral impairment are often studied later in the disease course than those with semantic impairment, because a loss of empathy may be misinterpreted as a psychiatric symptom, delaying their neuropsychological assessment. A very similar interpretation was given by Ulugut et al. [36], who suggested that, in patients with behavioral disorders, impaired face recognition may not be considered as a relevant problem by patients and caregivers, because specific tests for face recognition are usually not administered in general practice. A last conjectural reason could be that the speed and severity of the disease’s evolution may be lower in patients with a ‘semantic’ than in those with a ‘behavioral’ variant, leading to a greater chance of identifying the modality-specific stage of recognition impairment typical of the ‘prosopagnosic–semantic’ forms of right FTD. The small number of patients gathered in the present review did not allow us to tackle this complex problem, but, to check our last hypothesis, the time elapsed from the onset of symptoms to the neuropsychological examination and the clinical severity of the disease were evaluated for each patient. These data were reported in the section titled ‘Clinical data’ in Table 1 as ‘time from onset’ and ‘disease severity’.

The analysis seems to show that the length of time elapsed from the onset of symptoms to their evaluation varied greatly in all groups of patients, but the disease severity was greater in the ‘behavioral’ than in the ‘semantic’ forms of right FTD, irrespective of the time elapsed from the onset of symptoms to their evaluation. It remains therefore difficult to explain why in individuals with a ‘semantic’ variant of right FTD, recognition disorders were initially modality-specific (concerning the faces of known persons more than the pictorial representations of other UEs) and evolved only at a later time to a multimodal semantic disorder, whereas in patients with a ‘behavioral’ variant, recognition defects systematically consisted of multimodal semantic disorders equally affecting their visual and verbal recognition of persons and other UEs. In any case, the small number of subjects suggests that we should consider these observations as hypothesis-generating rather than confirmatory, as shown in the present review.

## 5. Conclusive Remarks

Some results of the present review were substantially consistent with models concerning the organization of knowledge and the steps of the recognition process within the right hemisphere, whereas other results were unexpected and require further investigations.

The first (expected) results obtained in patients diagnosed as having ‘semantic variants’ of right FTD was that information on the right hemisphere is based on the progressive post-perceptual processing of faces (and other non-verbal information). This process leads at first to the construction of modality-specific representations and then to the construction of multimodal semantic representations. The pathological correlates of these two processing steps after right ATL lesions are, respectively, the generation of ‘associative forms of prosopagnosia’ (e.g., [48,49,50]) and of semantic forms of familiar people recognition (e.g., [9,32,33]). The results of the present review confirmed that, in patients characterized by disorders of person recognition, it is possible to distinguish two groups of individuals. In patients in the first group (with prosopagnosic–semantic variants of FTD), recognition disorders are modality-specific and mainly concern familiar faces, without affecting or only slightly affecting other kinds of UEs, whereas in patients in the second group (with ‘properly semantic’ variants of FTD), recognition disorders are multimodal and equally affect faces and names of famous people and of other UEs.

The second (unexpected) result was that, in patients diagnosed as having ‘behavioral variants’ of right FTD, no progression was observed between modality-specific and properly semantic recognition disorders and that multimodal recognition disorders equally affected familiar people and other kinds of UEs. Apparently, the length of time elapsed from the onset of symptoms to their evaluation was not different in patients diagnosed as ‘semantic’ and as ‘behavioral variants’, whereas the overall severity of the disease was greater in the latter group. However, the small number of patients included in the review (which constitutes the main weakness of this study) did not allow us to evaluate the importance of this factor. In any case, the dissociations between patients with ‘prosopagnosic–semantic’ and with ‘properly semantic’ variants of right FTD and between the early stages of the ‘semantic’ and of the ‘behavioral’ variants of right FTD strongly suggest that we should pay more attention to the development and the time evolution of cognitive disorders in patients with ‘semantic’ and ‘behavioral’ variants of this disease. Better control of this variable could indeed allow us to evaluate if the differences noted between these two variants of right FTD are due to a bias in the stage of disease evolution in which these cognitive disorders are investigated or are due to intrinsic mechanisms leading more quickly to multimodal semantic disorders in patients with a ‘behavioral’ than in those with a ‘semantic’ right variant of FTD. Some longitudinal studies (e.g., [51,52]) have investigated the first symptoms of and the subsequent clinical evolution in patients with left versus right variants of FTD. These studies, however, have usually investigated the development of behavioral disorders or the shifts between defects of person recognition and behavioral disorders more than the development of different stages of person recognition disorders in patients in different diagnostic groups of right FTD. This aspect of the disease’s evolution constitutes the issue on which further investigations aiming to clarify the results of the present review should be focused.

## 6. Limitations of the Review and Reasons for These Limitations

The methodological limitations of and theoretical objections to the present review should be addressed. The first ‘caveat’ is that the review is based on a very small number of heterogeneous historical single-case reports, which span different decades, diagnostic frameworks, neuropsychological methodologies, imaging techniques, and stages of disease progression. A counter argument that can be raised to this objection is that several classic and contemporary authors (e.g., [53,54,55]) have suggested the use of single-case studies when the aim of an investigation is to clarify the nature of the mechanism underlying a target behavioral defect (in this case of ‘familiar people recognition disorders’). Group studies of patients presenting a target disturbance could indeed be heterogeneous in regards to the nature of the underlying mechanisms, whereas well-conducted single-case studies can be more homogeneous and informative on this subject. Authoritative classical researchers (e.g., [53,54]) have even recommended the use of ‘multiple single-case studies’ (very similar to the review of well-conducted single-case studies used in the present investigation) to carry out these kind of inquiries. Furthermore, the interest of single-case studies in the assessment of prosopagnosia has been recently suggested by an authoritative scholar of this discipline [55]. As mentioned in the introduction, the decision to carry out this review was due to the fact that many interesting single-case studies aiming to explore the functional architecture of familiar people recognition disorders were published many years ago, before neuroscientists were interested in these disorders, which are a pathognomonic feature of the right semantic variant of FTD. Therefore, since very few single-case reports with these characteristics have been published in more recent times, I thought that a review of these old studies could contribute to clarifying the theoretical aspects of the complex symptomatology presented by patients with a right variant of FTD [36].

A second objection might be raised in that the manuscript tends to treat the reviewed patients as belonging to relatively homogeneous clinical entities, when they likely represent a mixture of heterogeneous syndromes. It is difficult to evaluate the appropriateness of this objection, due to the complexity of the emotional, behavioral, and cognitive disorders observed in these patients (e.g., [27,28,34,36,47]). The small number of patients included in the present review decreased further, as they were fragmented into smaller groups to increase the homogeneity of the matched samples, but part of the intrinsic heterogeneity of the right variant of FTD probably lies in the complexity of the mnesic, perceptual, cognitive, emotional, and visceral functions subserved by adjacent right ATL structures and discussed in all of the above-mentioned general reviews.

The complexity of these functions and of their interactions can also at least in part account for another objection that could be raised to the present review, namely the lack of clear relations between the anatomical distribution of atrophy and the clinical form of RvFTD or the frequency of recognition disorders for famous persons and other UEs. In regards to this objection, it must in any case be acknowledged that the absence of clear anatomo-clinical correlations was mostly be due to the old imaging methods used in the different case studies, which varied considerably across reports and included MRI, SPECT, PET, and descriptive radiological assessments and to the fact that, in several cases, the imaging descriptions were not very clear.

## Data Availability

Data refer to new data obtained in original reviews, not to data gathered in reviews.
